# The Importance of Intratumoral Venous Drainage Preservation in Two-Stage Surgery of Large Hypervascular Choroid Plexus Papilloma: A Case Report

**DOI:** 10.7759/cureus.45796

**Published:** 2023-09-22

**Authors:** Daishiro Abe, Kohei Kanaya, Takafumi Kiuchi, Sumio Kobayashi, Tetsuyoshi Horiuchi

**Affiliations:** 1 Neurosurgery, Shinshu University School of Medicine, Matsumoto, JPN; 2 Neurosurgery, Iida Municipal Hospital, Iida, JPN

**Keywords:** complication, choroid plexus papilloma, venous drainage, surgical strategy, hypervascular tumor

## Abstract

Two-stage surgery may be necessary when total tumor removal cannot be accomplished in the first surgery; however, the extent and condition in which the remaining tumor should be before the next surgery have not yet been established. There is a risk of postoperative hemorrhage in the residual tumor, especially in hypervascular tumors.

We report a case of hypervascular choroid plexus papilloma (CPP) in a 22-year-old male patient where the preservation of intratumoral venous drainage was considered important to avoid hemorrhagic complications during a two-stage surgery. In the first surgery, it was difficult to control the bleeding from the debulked tumor, and the surgery was terminated due to severe blood loss. Large draining veins running in the tumor were preserved as it was suspected that these were important drainage routes of the bloodstream of the tumor. The preserved draining red veins changed to normal venous color in the second surgery performed after one week. The residual tumor was not vascularized during the second surgery and underwent gross total resection with less blood loss. The patient was discharged without sequelae. There was no recurrence of the tumor and no neurological deficit during the three-year follow-up. To prevent postoperative hemorrhage associated with a residual tumor, it may be important to preserve venous drainage of the tumor in hypervascular tumor resection.

## Introduction

Choroid plexus papillomas (CPPs) are rare indolent neuroepithelial tumors with high vascularity that arise from the choroid plexus [1]. Although the cornerstone of the treatment for CPPs is safe surgical resection [2,3], there is an increased risk of serious hemorrhage during surgery, which complicates surgical resection and limits the complete resection of these tumors [2]. Two-stage surgery should be considered based on intraoperative findings and characteristics of the tumor, such as tumor vascularity, operative blood loss, tumor adhesion [4,5], and surgical time. However, it has not yet been established how to leave the tumor for the next surgery when total tumor removal cannot be accomplished during the first surgery.

Surgical management of intracranial hypervascular tumors has always presented a substantial challenge to neurosurgeons due to intractable intraoperative hemorrhages [6]. Surgical strategies for hypervascular tumors have been described, mainly with regard to arterial processing [2]. Preserving arterialized venous drainage in vascular tumors is considered important in brain tumor surgery, which has rarely been reported in the literature [7]. We report a case of CPP in which resection by considering the preservation of venous drainage was important to avoid hemorrhagic complications during two-stage surgery. The current case indicates that it may be important to consider venous drainage preservation during tumor resection, especially in cases of hypervascular tumors.

This article was previously posted to the Preprints.org server on June 23, 2023.

## Case presentation

**Figure 1:**
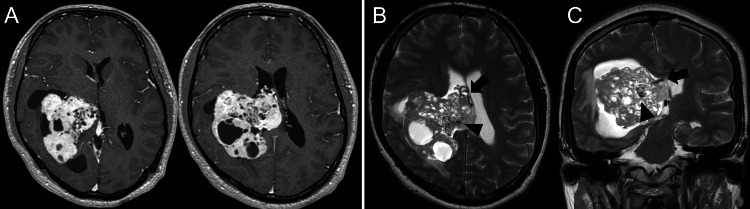
Preoperative MRI showing large hypervascular intraventricular tumor Contrast-enhanced T1-weighted axial image showing an enhancing multicystic tumor in the right lateral ventricle (A). T2-weighted axial and coronal images showing dilated intratumoral veins (black arrowheads) and large drainage veins running into the deep drainage system (black arrows) (B, C) MRI: magnetic resonance imaging

**Figure 2:**
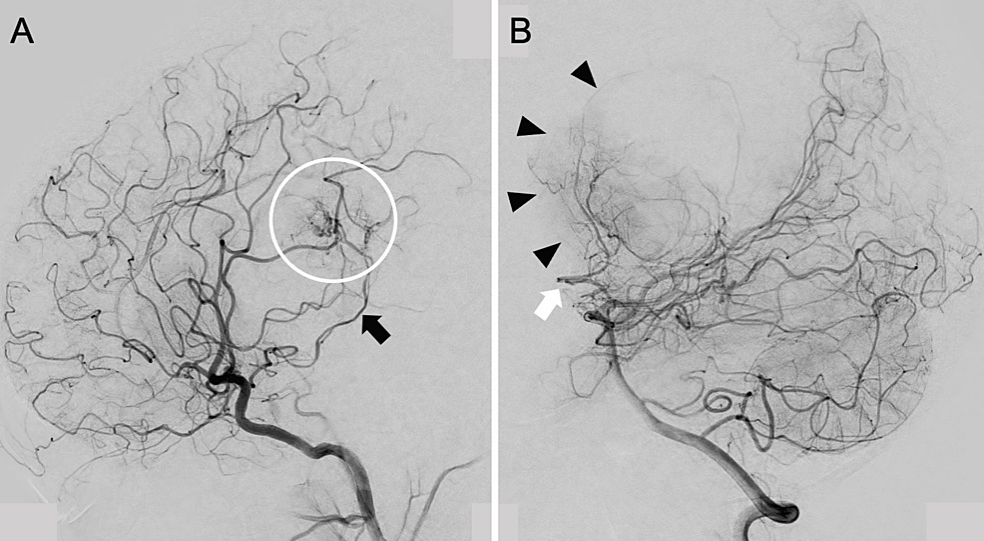
Preoperative angiogram showing multiple feeders and arterial-venous shunt in the tumor Right internal carotid angiogram showing that the feeder was the right anterior choroidal artery (black arrow) and arterial-venous shunt of the tumor (white circle) (A). Vertebral angiogram showing that the feeder was the right lateral posterior choroidal artery (white arrow) and tumor stain (black arrowheads) (B)

A 22-year-old right-handed healthy male presented with chief complaints of progressive headache and nausea that had persisted for two weeks. Neurological examination revealed no abnormal findings except for increased intracranial pressure signs including headache and nausea. Contrast-enhanced MRI showed a well-defined, highly enhancing multicystic tumor extending from the inferior horn to the body of the right lateral ventricle (Figure 1A). Marked dilated intratumoral veins and peritumoral brain edema were observed in the right temporal and parietal lobes, associated with a slight midline shift to the left, and the inferior horn of the right lateral ventricle was entrapped (Figures 1B, 1C). Cerebral angiograms revealed that the tumor was fed by the anterior and lateral posterior choroidal arteries (Figures 2A, 2B). Preoperative endovascular feeder embolization with successful flow reduction using coils in the anterior choroidal artery was achieved a day before the removal surgery.

**Figure 3:**
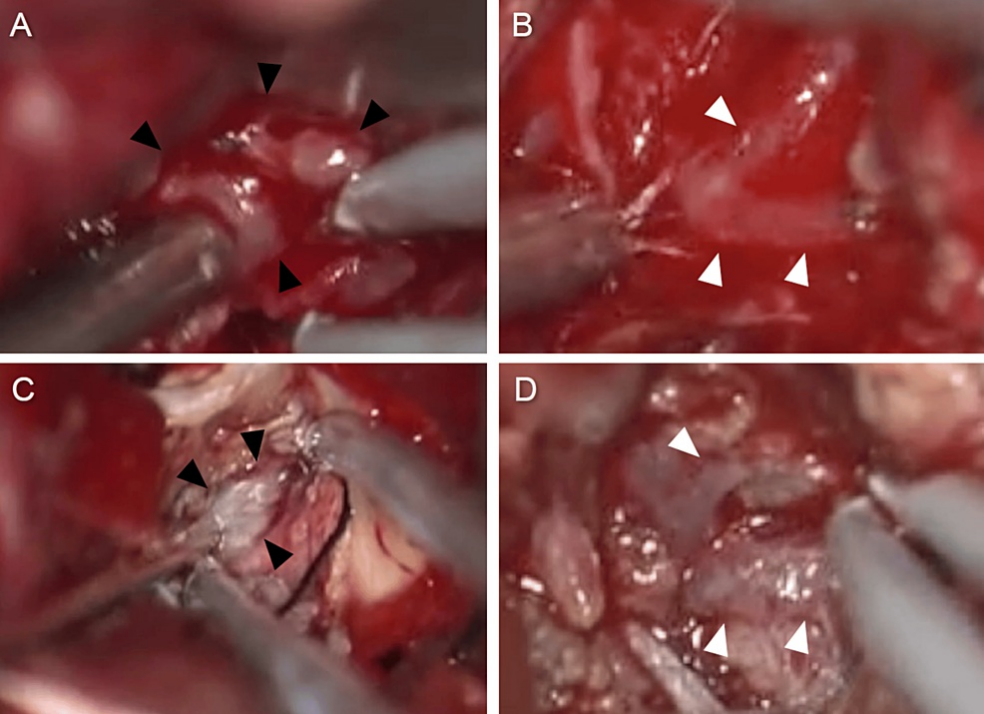
Intraoperative images during the first and second surgeries Intraoperative images during the first surgery show severe bleeding during tumor resection (A). The dilated red vein in the tumor (white arrowheads) in the first surgery (B). Intraoperative images in the second surgery show less bleeding during tumor removal (C). The red vein in the first surgery turns to the normal color (white arrowheads) in the second surgery (D). Black arrowheads indicate the tumor (A and C)

Tumor removal was planned. Under general anesthesia, a right temporal craniotomy was performed. A 2-cm cortical incision in the middle temporal gyrus and a trajectory into the inferior horn of the lateral ventricle were made. A reddish tumor that bleeds easily was observed in the ventricle (Figure 3A). The tumor was elastic, soft, and suckable. The lateral part of the tumor was resected and dissected from the ventricles. It was difficult to control the bleeding from the debulked tumor; therefore, removal of the medial part of the tumor was considered risky. The surgery was terminated due to severe blood loss (1732 mL) and prolonged operative time (530 minutes). Large draining veins running in the tumor were not coagulated and preserved because they were red veins and it was suspected that they were important drainage routes of the bloodstream of the tumor (Figure 3B).

**Figure 4:**
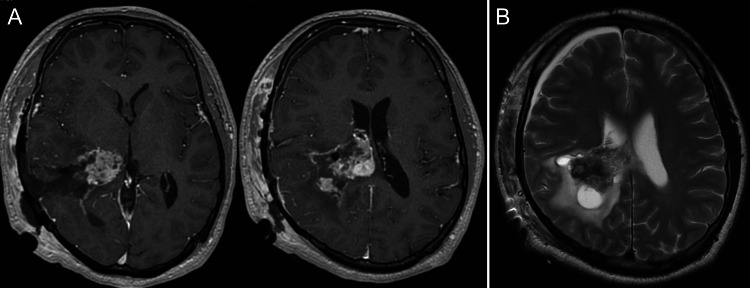
MRI images on postoperative day 5 showing a decrease in tumor enhancement and disappearance of the intratumoral dilated veins Contrast-enhanced T1-weighted axial image on postoperative day 5 showing partial removal of the tumor, and a decrease in enhancement of the residual tumor (A). T2-weighted axial image on postoperative day 5 showing the disappearance of the intratumoral dilated veins that were seen on the initial MRI (B) MRI: magnetic resonance imaging

MRI performed five days after the surgery showed a residual tumor with decreased enhancement and disappearance of the intratumoral dilated veins, suggesting that the blood supply to the tumor was reduced (Figures 4A, 4B). Increased intracranial pressure signs including headache and nausea did not improve after the first surgery; therefore, the second surgery was planned.

**Figure 5:**
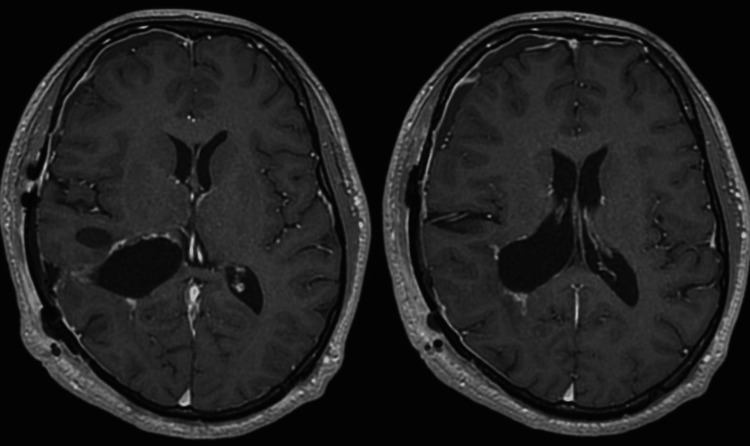
Postoperative MRI after second surgery showing gross total removal of the tumor Contrast-enhanced T1-weighted image showing the adequate removal of the tumor MRI: magnetic resonance imaging

Postoperative MRI indicated a decline in tumor vascularity, and an increase in the distance from the parietal lobe to the lateral ventricle due to the partial removal of the tumor and a decrease in the enlarged ventricle; therefore, the second surgery was performed using the same approach after one week. The residual tumor was yellowish and not vascularized compared to the prior appearance of the tumor during the first surgery (Figure 3C). The draining red veins preserved in the first surgery changed to a normal venous color (Figure 3D). The tumor underwent gross total resection, with a total blood loss of 325 mL. MRI performed one week after the second surgery showed that the tumor had been almost completely removed (Figure 5). The patient tolerated the treatment well, and his intracranial pressure signs such as headache and nausea improved.

The pathological diagnosis was grade I CPP based on the World Health Organization classification of tumors of the central nervous system [8]. There was no evidence of malignancy. The pathological findings of the tumor were not different in both surgeries. The patient was subsequently discharged without sequelae. There was no recurrence of the tumor and no neurological deficit during the three-year follow-up.

## Discussion

The extent and condition in which the remaining tumor should be before the next surgery have not yet been established when total removal of the tumor cannot be accomplished in the first surgery, especially in hypervascular tumors. Surgical strategies for hypervascular tumors are often described with regard to arterial processing (e.g., preoperative endovascular feeder embolization) [2,9]. However, only a few studies have focused on venous drainage [7]. We hypothesized that it is important to preserve venous drainage of the tumor in hypervascular tumor resection to prevent postoperative hemorrhage associated with a residual tumor.

Choroid plexus tumors are usually highly vascularized, and intraoperative hemorrhage is a serious risk, especially in pediatric patients [3]. These tumors develop in the ventricles; therefore, tumor-related hemorrhage can directly induce intraventricular hemorrhage, which is associated with high mortality and morbidity [10]. It is critical to prevent tumor-related, intraoperative, and postoperative hemorrhages when manipulating CPPs.

In this case, CPP was considered a hypervascular tumor on preoperative imaging. In terms of arterial processing, preoperative endovascular feeder embolization of the right anterior choroidal artery was performed. Although it may have been difficult to deal with the blood supply from the posterior choroidal feeders, we chose the middle temporal gyrus approach because of the location of the tumor and the ease of approaching the lesion. The same approach was chosen in the second surgery and not the high parietal approach. The enlarged ventricle became smaller after the first surgery, which indicated that the distance from the parietal lobe to the lateral ventricle became longer. Furthermore, MRI after the first surgery showed a decrease in enhancement and a decrease of the draining vein of the tumor, which indicated that the vascularity of the tumor had declined compared to the first surgery. 

Because of the arteriovenous malformation-like nature of hypervascular tumors, loss of venous drainage would be considered to increase the risk of intratumoral hemorrhage [7]. Venous drainage of the tumor should be considered and preserved when the tumor cannot be completely removed to prevent postoperative hemorrhage related to disruption of venous drainage. Particularly, the red vein is expected to be the primary drainage pathway for the tumor, reflecting the abundant feeder blood flow to the tumor and a high number of arteriovenous shunts. In the present case, intentional preservation of the red vein may have contributed to venous drainage, preventing postoperative hemorrhage caused by venous disruption of the tumor.

MRI on postoperative day five showed a decrease in tumor enhancement and disappearance of the intratumoral dilated veins (Figure 4). Furthermore, the red vein observed in the first surgery changed to a normal color in the second surgery (Figure 3). Our result indicates that MRI should be confirmed before the second surgery in order to devise a proper surgical strategy as MRI can predict the vascularity of the tumor [11]. In this case, the color tone did not normalize during the first surgery; however, this was confirmed during the second surgery after one week. It is suggested that hemodynamic changes in the tumor take a few days. Yamakami et al. and LeMay et al. found that tumor blood flow was reduced after internal decompression [9,12]. In the present case, the tumor tissue obtained from the first and second surgeries showed no differences in pathological examination. We speculated that preoperative feeder embolization and partial tumor resection may have resulted in a reduction in blood flow, leading to reduced bleeding in the second surgery. The mechanism of alteration of the blood supply to the residual tumor remains unknown. Hence, further studies are necessary to elucidate the alterations in the blood supply and venous drainage of hypervascular tumors. 

Postoperative hemorrhage can be classified as arterial- and/or venous-related hemorrhage. Generally, blood pressure control is important to prevent postoperative hemorrhage [13]. Arterial-related postoperative bleeding can be prevented by controlling the blood pressure; however, it can be difficult to prevent venous-related hemorrhage from residual tumors. We speculated that the partial removal of the tumors, especially hypervascular tumors, may itself be considered a risk factor for postoperative venous-related bleeding. Postoperative venous-related hemorrhage related to the residual tumor can be caused by hemodynamic changes, especially a decrease in drainage pathways. Therefore, it is important to preserve venous drainage to prevent postoperative venous-related hemorrhage when surgeons choose to leave the tumor during surgical removal, which has not been reported before to the best of our knowledge.

This report has several limitations. Firstly, this is a clinical case report. Secondly, it is difficult to evaluate the required amount of venous drainage for residual tumors during surgery. Moreover, it can be difficult to predict the relationship between postoperative venous-related hemorrhage from residual tumors and the preservation of the intratumoral red veins. Further research is necessary to elucidate the exact evaluation of the venous drainage of the tumor. Despite these limitations, this case report highlights the importance of intratumoral venous drainage preservation in two-stage surgery.

In the present case, two-stage surgery was successfully performed for a highly vascularized CPP without any complications. To prevent postoperative complications, especially postoperative hemorrhage associated with residual tumor, a detailed preoperative evaluation of the tumor, involving not only arterial but also venous hemodynamics of the tumor, is required. Furthermore, it is important to orient intraoperative findings by considering tumor vascularity, intraoperative bleeding, and venous drainage of the tumor when it is left out.

## Conclusions

We reported a case of large hypervascular CPP treated with two-stage surgery by considering the preservation of intratumoral venous drainage, without any complications. Residual tumors, especially hypervascular tumors, may carry a risk of postoperative tumor-related hemorrhage. Preservation of intratumoral venous drainage can be important to prevent postoperative venous-related hemorrhage when total removal of the tumor is not performed. Intentional preservation of venous drainage, especially red veins, may contribute to preventing postoperative hemorrhage caused by venous disruption of the tumor. Large-scale studies are required to further elucidate the importance of the venous drainage of hypervascular tumors, and further research involving quantitative analyses of the tumor blood flow, not only for arterial supplies but also for venous drainages, is warranted.

## References

[1] Safaee M, Clark AJ, Bloch O (2013). Surgical outcomes in choroid plexus papillomas: an institutional experience. J Neurooncol.

[2] Dash C, Moorthy S, Garg K (2019). Management of choroid plexus tumors in infants and young children up to 4 years of age: an institutional experience. World Neurosurg.

[3] Safaee M, Oh MC, Bloch O (2013). Choroid plexus papillomas: advances in molecular biology and understanding of tumorigenesis. Neuro Oncol.

[4] de Melo Junior JO, Landeiro JA, Leal da Silveira R (2023). Large vestibular schwannoma: a two-stage surgery. Cureus.

[5] Raslan AM, Liu JK, McMenomey SO, Delashaw JB Jr (2012). Staged resection of large vestibular schwannomas. J Neurosurg.

[6] Li P, Tian Y, Song J (2021). Microsurgical intracranial hypervascular tumor resection immediately after endovascular embolization in a hybrid operative suite: a single-center experience. J Clin Neurosci.

[7] Rachinger J, Buslei R, Prell J, Strauss C (2009). Solid haemangioblastomas of the CNS: a review of 17 consecutive cases. Neurosurg Rev.

[8] Louis DN, Perry A, Wesseling P (2021). The 2021 WHO Classification of Tumors of the Central Nervous System: a summary. Neuro Oncol.

[9] LeMay DR, Sun JK, Fishback D, Locke GE, Giannotta SL (1998). Hypervascular acoustic neuroma. Neurol Res.

[10] Hinson HE, Hanley DF, Ziai WC (2010). Management of intraventricular hemorrhage. Curr Neurol Neurosci Rep.

[11] Villanueva-Meyer JE, Mabray MC, Cha S (2017). Current clinical brain tumor imaging. Neurosurgery.

[12] Yamakami I, Kobayashi E, Iwadate Y, Saeki N, Yamaura A (2002). Hypervascular vestibular schwannomas. Surg Neurol.

[13] Basali A, Mascha EJ, Kalfas I, Schubert A (2000). Relation between perioperative hypertension and intracranial hemorrhage after craniotomy. Anesthesiology.

